# Place of residence and coach-athlete relationship predict drop-out from competitive cross-country skiing

**DOI:** 10.3389/fspor.2023.1110060

**Published:** 2023-04-19

**Authors:** Marit Anita Schmid, Guro Strøm Solli, Rune Kjøsen Talsnes, Frode Moen

**Affiliations:** ^1^ Department of Education and Lifelong Learning, Faculty of Social and Educational Sciences, Norwegian University of Science and Technology, Trondheim, Norway; ^2^ Department of Sports Science and Physical Education, Nord University, Bodø, Norway; ^3^ Centre for Elite Sports Research, Department of Neuromedicine and Movement Science, Faculty of Medicine and Health Science, Norwegian University of Science and Technology, Trondheim, Norway

**Keywords:** youth sports, drop-out, cross-country skiing, elite sport, coach-Athlete relationship

## Abstract

The current study investigated whether factors such as living residence, the coach-athlete working alliance, goal orientation, and intrinsic motivation could explain drop-out, and whether these factors differed between athletes included in the elite- vs. general sport programs during high school years. In total 257 cross-country skiers, graduated from three different Norwegian Top Sport schools during the period from 2015 to 2019, were recruited to retrospectively investigate their experiences related to the time period when they participated in programs for cross-country skiing at high school. In total 116 of these athletes completed an online survey including validated and standardized instruments for the assessment of the coach-athlete working alliance (CAWAI), goal orientation (GO), perceived satisfaction with their performances (PAP) and intrinsic motivation (IM). The results showed that 84% of the athletes had dropped out from cross-country skiing, while 16% were still active. The highest ranked fixed statements of causes for drop-out was “*a natural choice*” (3.79 ± 1.11), “*priority of education or work*” (3.61 ± 1.30), “*lack of motivation*” (3.49 ± 1.28), “*negative performance development*” (3.46 ± 1.18), and “*challenges with health*” (3.25 ± 1.54). There were significant differences between active and drop-out in scores for reciprocity between the coaches' and the athletes' perceptions of goals (CAWAI-goal; 5.87 ± .98 vs. 5.07 ± 1.15; *p* = .004), the coach-athlete relationship bond (CAWAI-bond; 6.08 ± .91 vs. 5.07 ± 1.38; *p* = .001), and tasks chosen to reach the defined goals (CAWAI-task; 5.61 ± .92 vs. 4.90 ± 1.09; *p* = .006). Furthermore, active athletes had higher mastery orientation (22.11 ± 2.88 vs. 20.00 ± 3.74; *p* = .010). A hierarchical binary logistic regression analysis showed that place of residence and the coach-athlete working alliance were significant predictors of drop-out while mastery and performance goal orientation or intrinsic motivation were not significant. All five predictors explained 23% of the variability in drop out from cross-country skiing. Overall, 58% and 42% of the athletes participated in the elite and general programs for cross-country skiing during high school, respectively. The athletes that took part in the elite programs reported significantly stronger coach-athlete working alliances (CAWAI-sum; 14.46±3.10 vs. 14.28±3.37; *p* = .000), higher mastery orientation (21.19±3.50 vs. 19.36±3.66; *p* = .008), and performance satisfaction (PAP; 19.17±5.98 vs. 15.69±5.19; *p* = .001) compared to the athletes attending general programs. The results are discussed in terms of existing knowledge on how place of residence, the coach-athlete working alliance, goal orientation, performances, and motivation might impact drop-out in cross-country skiing.

## 1. Introduction

The importance of engaging in sport and physical activity has received much attention in research over the last decades (1, 2). Research claims that regardless of type of sport (team or individual), age, and somatic or mental health problems, there is consistent evidence that engagement in sport and physical activity is positively associated with social-, physical- and psychological health (1). Further, there is evidence suggesting that the positive associations between participation in sport and physical activity are stronger in team sports than in individual sports (1). Taken together, participation in sport and physical activity is found to be positively associated with good health.

However, over the past decades, research has found that drop-out from sport and physical activity increases from childhood to adolescence (3–6), and to early adulthood (7). As researchers have concentrated more on the phenomenon of drop-out in a sport-specific context, their attention has been on a variety of different sports (4, 8). The majority of previous studies have focused their attention on children and early adolescents who may not have specialized in a single sport yet (8). There are also several studies that have not disclosed which sport context they examined (4). Herein, there is currently limited knowledge concerning individual endurance sports such as cross-country skiing specifically (9). Most previous studies in cross-country skiing have examined physiological, biomechanical and psychological factors and their corresponding associations to performance (10). Nonetheless, one study investigated drop-out from cross-country skiing and the findings aligned with the previous research on drop-out in sports in general, which claims that high sport specific demands, failure of reaching own goals, and spending time on other activities were the most reported reasons for drop-out (9). With cross-country skiing being a highly demanding endurance sport, the average ages (27.0 years for females and 29.0 years for males) of international elite skiers are relatively high (11). In a more recent study, Walther and colleagues (12) reported the peak ages for international cross-country skiers to be 26.2 years for the distance events and 26.0 years for the sprint events. Considering that previous research have shown high numbers of drop-out from sport and physical activity before the age of 25.0 (4, 13, 14), there are many athletes that end their athletic careers before reaching the age of peak performance in their respective sport. Therefore, the current study will focus on which factors that may determine drop-out from competitive cross-country skiing during late adolescence, and thereby explore potential actions to reduce drop-out in cross-country skiing.

The term drop-out has been used interchangeably in research with “attrition”, “withdrawal”, “retirement”, “disengagement”, “opting-out” and “sport discontinuation” (4, 13–15). The definition varies from full discontinuation from all sports and physical activity, to change from one sport to another (talent transfer), and leaving sports for an unspecified period (13, 15). The current study will use the term “drop-out” and the definition will include an athlete's discontinuation from competitive sports.

The phenomenon of drop-out from sports first became a topic of interest in research during the seventies, and then became increasingly important in the eighties (15). To summarize the research, drop-out can be explained by intrapersonal, interpersonal and structural constraints (8). Intrapersonal and interpersonal constraints were the far most reported variables compared to structural constraints such as time, injuries, cost and inadequate facilities (16, 17). Pressure, other social priorities, motivational factors (18, 19) and having other things to do were the most reported interpersonal constraints, but lack of support from parents, teammates or coaches (18, 20–22) are also found to be associated with drop-out. Intrapersonal constraints such as lack of enjoyment or interest in the sport (6), perception of physical competence and intrinsic pressure (18, 21), were the most reported intrapersonal constraints that were associated with drop-out from sports. The most dominant constraints, which were associated with drop-out in 26 separate studies out of the 43 included in the review, were lack of enjoyment of sport and athletes' perceptions of their own sport specific skills (8). What contributed to lack of enjoyment is not fully explored, but factors such as not having enough playing time or opportunities, dissatisfaction with the coach and too much training time were reported as reasons that led to lack of enjoyment.

The current study will further focus on the two most reported causes of drop-out in competitive sports, the intrapersonal constraint such as perception of physical competence, and interpersonal constraints such as the coach-athlete relationship, goal-orientation, and intrinsic motivation.

A recent study found that 80% of the athletes who attend to Norwegian high schools specialized for elite sports had ambitions to become future elite athletes in their respective sports (23). The athletes' perceptions of their sport specific skills and capacities are associated with the feeling of being good enough compared to their ambitions as athletes, and the degree of skill improvement in their sport (8). In general, athletes' ambitions are found to be high, and their ambitions will affect their perceptions of their physical competence. Thus, distance between ambitions and the athletes' actual physical competence over time will negatively affect their perceived physical competence, and when athletes' perceptions of their own competence are lower, their motivation are also lower (24). Therefore, the association between athletes' perception of their physical competence and drop-out is logical (8). However, there is an expectation among athletes that their coaches possess abilities to help promote the development of their needed skills and capacities in their sports (25). Thus, coaches in sport are responsible for establishing effective helping relationships with their athletes that ultimately grow their talents towards their ambitions (26). To succed in developing their athletes coaches need to have competencies that enable them to build strong coach-athlete relationships. The coach-athlete relationship is therefore an important factor that are also associated with drop-out in sports (18, 20–22).

Research has emphasized the importance of an effective coach-athlete relationship in order to affect performance (27), as well as the motivation and athlete's development and well-being (28–30). Being in an elite competitive and training environment requires a lot from an athlete, especially in the transition from junior to senior years. Navigating such an environment for athletes would ideally be assisted by effective coaches, helping them both in navigating the competitive landscape and developing buffers against the possible negative effects of other stressors they encounter outside of the competition context (31). Previous research has suggests coaches' psychological and pedagogical abilities, expert qualifications and competence, and knowledge in sport-specific training are crucial for enhancing the athletes' performances and avoiding loss of their motivation (32). Several theoretical frameworks have been developed to study the coach-athlete relationship, including, among others a motivational model (33) and a relational model (the 3C's + 1 (34);. One measure that is used to investigate this relationship is the Coach-Athlete Relationship Questionnaire (CART-Q), which applies the concept of the 3C's (35). Another measure is the Coach-Athlete Working Alliance Inventory [CAWAI; (23, 36)].

The CAWAI was developed based on four different theories; Carl Rogers' client-centered approach, Strong's social influence theory, the psychodynamic perspective on working alliance, and Bordin's (37) theory on working alliance (38). Bordin (37, p. 253) combined aspects of psychoanalytic literature to present his three key aspects of the working alliance: an agreement on goals, an assignment of a task or series of tasks to reach goals, and the development of bonds. The primary objective of the original Working Alliance Inventory (WAI) was to measure some of the variables affecting the degree of success, or non-success, in counseling based on the working alliance framework (38). As Moen, et.al. (23) clarified, the coach-athlete relationship setting differs from the therapeutic setting, however both settings require a helping relationship built on a close and trusting bond, and an agreement about goals and tasks to reach these goals. Previous research on the working alliance in the sport context (CAWAI) show that the inventory is positively associated with performance satisfaction and negatively associated with an athlete's worry and negative affect (28). One study investigated possible associations between coaches’ personalities and the coach-athlete relationship (39) and reported significant associations between extraversion and grit, and the CAWAI. Thus, the process of athlete development involves coaches' abilities to establishing a strong bond with their athletes, a clarity of their goals and the corresponding tasks required to reach their goals.

Goal orientation has been researched in connection with performance in the workplace- (40, 41), school- (42), and in the sport setting (43). Goals can be defined as clearly formulated thoughts, ideas or intentions about a specific valued and desired outcome, which a person, an environment or an organization are attempting to achieve within a set time period (44), and are a standard by which to evaluate performance (40). Goals can be grouped into types, or categories, and a commonly used sport-categorization is: performance goals and mastery goals (42, 43, 45, 46). Performance goals usually focus on an outcome, such as competition results, and/or an individual's performance in relation to a normative standard, and mastery goals focus on how a skill, technique or strategy is performed, and on the development and process of learning the skill itself is valued by the individual. With mastery goals, an individual seeks to improve competence by learning or mastery and rates performance by one's own standard (42, 45, 46).

Goal orientation is an underlying motivational factor that influences an individual's goals and desire to demonstrate ability and become successful, and research generally distinguishes between two types of goal orientation: task orientation and ego orientation (43, 47). Task orientation refers to the attention towards the learning and development process itself, and improving understanding, skills, attitudes, and mastering tasks. Ego orientation focuses on the person in the development process and the goal is to appear successful or avoid appearing unsuccessful. Performance goals are typically defined as ego oriented, while mastery or process goals can be considered as task oriented. Task, or mastery, orientation has been shown to be most favorable for performance (43), whereas ego, or performance, orientation seems to be associated to higher risk of drop-out (48). Importantly, these orientations are orthogonal and individuals can be high or low in each, or both orientations at the same time (43, 49).

Furthermore, the achievement goal theory claims that when an individual performs achievement-related tasks they can fluctuate in their state of involvement directed towards task (mastery) or ego (performance) goals (49). The theory is based on the idea that variations in perceptions of competence or ability and how one defines successful accomplishments are crucial antecedents for understanding an athlete's motivational processes.

Motivation can be divided into two main types: intrinsic motivations and extrinsic motivations. Intrinsic motivation involves doing something for one's own sake, while extrinsic motivation involves doing something to earn a reward or avoid punishment. Self-determination theory states individuals need to be autonomous, engage in activities they desire and make decision about how to act. Perceived control is an important determinant of intrinsic motivation and, in antithesis, our motivation suffers when people cannot exercise self-determination (50).

Multiple previous studies have investigated motivational factors in relation to performance and drop-out from sports (19, 51, 52). More specifically, Enoksen (32) found general motivation to be a factor for drop-out, while Jõesaar and Hein (18) and others (52) found intrinsic motivation, specifically, to be more closely related and more important to further continuation in the sport. As stated in the definition of goal orientation above, the orientation is a motivational factor of goals. Motivation has been thoroughly researched, and studies have investigated motivation within the context of school, work, sport, and other areas. Schunk, Meece and Pintrich (53, p. 5) define motivation as “the process whereby goal-directed activities are instigated and sustained”.

The current study aims to investigate if the coach-athlete working alliance, goal orientation, and intrinsic motivation can explain drop-out from sports among Norwegian cross-country skiers. The abovementioned theoretical framework has focused on physical competence, the coach-athlete working alliance, goal orientation and intrinsic motivation. The authors have concentrated on these factors due to the presented research findings and the potential association to drop-out in sport-specific contexts. Previous research have suggested that athletes are less likely to drop-out from sports when they have strong parental, or familial support (18). Therefore, the current study also includes residence situation as a variable of interest, as some high school students in Norway move away from their families to attend schools and live in a residence away from their family residence. Therefore, the current study examined whether Norwegian cross-country skiers who continued with competitive cross-country skiing after graduating from high school had a stronger working alliance with their coaches, higher mastery- and performance goal orientation, higher motivation and performed better than cross-country skiers who had dropped out from competitive cross-country skiing. Furthermore, the current study aims to examine if place of residence, the coach-athlete working alliance, mastery- and performance goal orientation, and motivation uniquely and collectively predict drop out from cross-country skiing. Lastly, the current study aims to investigate if elite groups in cross-country skiing at high schools have stronger coach-athlete working alliances, higher mastery and performance goal orientations, higher motivation and perform better than general cross-country skiing groups.

## 2. Method

Public Norwegian high schools for elite sports provide an unique opportunity for young athletes who have ambitions to succeed in their sports to develop their potential as athletes. Systematic training and professional help and support from competent coaches are a key part of the athletes' educational plan in such high schools. In order to investigate how the coach-athlete working alliance, goal orientation and intrinsic motivation are uniquely associated with the potential for cross-country skiers to drop-out from their sport, a cross-sectional design was utilized to investigate the thoughts and opinions from athletes that had graduated from Norwegian high schools offering cross-country skiing as one of their sports. Three different public high schools specialized for sports in Norway were contacted and informed about the aim of the study and asked to participate. The three high schools were the only schools in Central Norway that offer both an elite program in cross-country skiing and a general program in cross-country skiing. All three high schools agreed to participate.

### 2.1. Participants

The participants in the current study were selected from the graduated classes from the years 2015–2019 from the three respective high schools. Both athletes who participated in the elite sport program- and the general program in cross-country skiing were selected to participate in the study. The athletes who are most talented based on their ambitions, mental-, physical- and social capacities and skills, are selected to elite sport programs during their high school years by the coaches responsible for those programs. Athletes who do not have such talents are chosen for the general programs. Of the total sample of 279 athletes graduated from the cross-country program (elite and general) during the selected time period, the researchers were able to receive contact information for 257 of these athletes. Thus, 257 graduated athletes were invited to participate in the study. The study was approved by the Norwegian Social Science Data Services and the participant provided informed consent to participate in this study.

Of the 257 athletes invited to participate, 116 completed the questionnaire (response rate = 45%), including 19 (16%) athletes that still competed in cross country skiing, and 97 (87%) that had dropped out. Fifty-eight athletes participated in the elite program for cross-country skiing during the time in high school, where 41 out of this group lived at a residence outside their family home and 17 lived at their family residence. Fifty-five athletes participated in the general program in cross-country skiing, where 35 out of them lived at a residence outside their family home and 20 lived at their family residence. Thirteen out of the athletes that participated in the elite program where still active (22%), while 6 athletes that participated at the general cross-country skiing program where still active (11%). Out of the nineteen athletes who were still active athletes, 17 lived at a residence away from their family during their time in high school (2 at family residence), and out of the 97 who had dropped out 60 lived at a residence away from their families (37 at family residence). Thirty of the athletes were females (26%), 36 were males (31%), and 50 did not report their sex (43%). Their ages ranged from 22 to 26 years old at the time of participation in the current study. The participants completed a mean ± standard deviation (SD) of 491 ± 99 training hours each year the first two years at the high school (minimum = 197 and maximum = 763), whereas they completed 542 ± 145 training hours in the last two years (minimum = 0 and maximum 850).

### 2.2. Procedure

A digital questionnaire was developed to collect information about the athletes' current status as athletes, and their experiences related to the time period when they participated in programs for cross-country skiing at the three high schools selected for the current study. The questionnaire was conducted online and took approximately 15–20 min to complete. Reminders were sent three times to non-responding participants over a period of 8 weeks. All the standardized inventories were translated from their original language to Norwegian by the authors.

### 2.3. Instruments

The digital questionnaire included questions about demographics such as age, sex, if they lived at a dorm during high school or at home in their family residence (place of residence), the annual training volume during the high-school years (the athletes normally register their training in training diaries during high school and they were asked to document their yearly training volume during the years at high school), if they are still active in competitive cross-country skiing, and standard instruments for the assessment of the coach-athlete working alliance, goal orientation, perceived satisfaction of their performances and intrinsic motivation. The standard instruments that were used in the current study are based on previously developed scales proven to hold both satisfactory validity and reliability (23, 50, 54, 55). The measurements are described below in more detail. The athletes were asked retrospectively, 2–6 years after graduating, to evaluate their time during high school and their experiences with the cross-country skiing program at their respective school. They were encouraged to reflect on the items based on this period in their life as sincerely and honestly as they could.

#### 2.3.1. Fixed causes to drop out from cross-country skiing

If the athletes had dropped out from cross-country skiing, they were asked to rate the reason why they dropped out based on fixed statements on a five-point scale ranging from “completely disagree” (1) to “completely agree” (5). The fixed statements were: high demands of equipment and economic resources, ineffective coach-athlete relationship, ineffective sports environment, not optimal training facilities, negative performance development, lack of variation in competition program, priority of education or work, lack of motivation, it was a natural choice, challenges with health (injuries and/or illness).

#### 2.3.2. The coach-athlete working alliance inventory (CAWAI)

The current study used a Norwegian version of the Coach-Athlete Working Alliance Inventory (CAWAI) to measure athletes’ perceptions of their relationships with their coaches (23). The Coach-Athlete Working Alliance Inventory (CAWAI) includes three separate subscales that measure the reciprocity between the coaches' and the athletes' perceptions of goals (CAWAI-goal), tasks chosen to reach the defined goals (CAWAI-tasks), and their relational bond (CAWAI-bond). The CAWAI is found to be significant associated with the competence coaches have to develop their athletes within their sports, and prevent that athletes experience non-functional states such as athlete burnout (23). The participants were asked to respond on 12 statements using a 7-point Likert scale, where four statements represent each subscale, and one statement in each subscale was reversed coded. The SUM-scale is calculated by adding the three subscales. Examples of questions from the CAWAI-bond subscale are, “There is mutual trust between the coach and athlete” and “The athlete is confident that the coach has knowledge that will be helpful”. Cronbach's alpha for the scale was .94 for the sum scale, .83 for the goal subscale, .82 for the task subscale, and .92 for the bond subscale.

#### 2.3.3. The perceived athlete performance (PAP)

An adjusted version of the “individual performance” dimension from the Athlete Satisfaction Questionnaire (ASQ) was used to measure the athletes' perceived satisfaction with their own performance development in their sports (55). This subscale seeks to measure the perceived satisfaction with progress in one's own task performance. Task performance includes a perception of absolute performance, improvements in performance, and goal achievement. An example of an item is: “I am satisfied with the development of my performance during this period.”. The athletes were asked to consider four items and how satisfied they were with their own progress as athletes in their sport on a 7-point scale ranging from 1 (“strongly disagree”) to 7 (“strongly agree”). The Cronbach's alpha for the scale was .94.

#### 2.3.4. Goal orientation (Go)

Goal orientation was measured based on Midgley et al.'s (56) operationalization of the concept, and goal orientation is divided into mastery orientation and performance orientation (54). The scale was originally developed to cover goal orientation in the school setting, and in the current study, the scale was adjusted to a sport-context version where words like class were changed to team, grades to results, and tests to competitions. Four items reflected each goal orientation and an example of item covering mastery orientation is: “What mattered in our team at school was to do our best.”, and performance orientation: “The most important thing was to achieve good results in competitions.” The scale was ranging from 1 (“strongly disagree”) to 7 (“strongly agree”). The Cronbach's alphas for the scale were .70 and .65 for mastery- and performance orientation, respectively.

#### 2.3.5. Intrinsic motivation (Im)

Intrinsic motivation was measured based on (50) and Vallerand et al.'s (57) scales. The scale includes four items ranging from 1 (“strongly disagree”) to 7 (“strongly agree”). An example of items covering intrinsic motivation is: “I really liked to be very active in my sport”, and “The requirements in my sport really interested me”. The Cronbach's alpha for the scale was .89.

**Figure 1:**
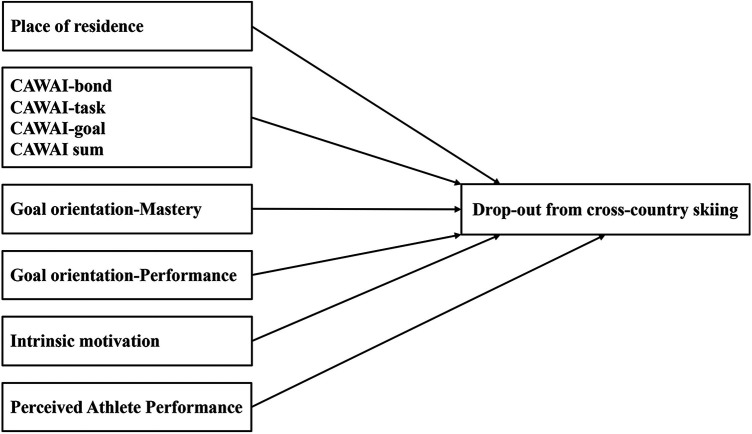
The variables in the study possible association to drop-out in cross-country skiing.

Figure 1 shows the variables in the current study and possible relations to drop-out from cross-country skiing.

### 2.4. Statistical analyses

Descriptive statistics, including means, SD, minimums, maximums and Cronbach's alpha coefficients for all variables in the model were calculated. Preliminary analyses were conducted to ensure no violation of the assumptions required to perform a hierarchical multiple regression analysis (normality, linearity, multicollinearity, and homoscedasticity).

Independent-sample t-tests were conducted to compare the groups of “active” vs. “dropped out” athletes and their scores on the different scales used in the study: CAWAI-bond, CAWAI-task, CAWAI-goal, CAWAI-sum, PAP, mastery orientation, performance orientation, and intrinsic motivation. Furthermore, independent sample t-tests were performed to compare the elite program and general program athletes with the aforementioned scale scores.

Finally, a hierarchical binary logistic regression analysis was calculated to predict if athletes were still active or had dropped out of cross-country skiing based on place of residence, the coach-athlete working alliance, mastery and performance goal orientation and intrinsic motivation. The CAWAI-sum variable was used in the regression analysis because of high covariance between the three subscales, and the subscale CAWAI-goals and the two GO variables. Thus, the CAWAI-sum variable provided the most significant model. The data for place of residence was transformed into numerical values where 1= “I lived at a dorm during high school”, and 2= “I lived at my family's residence during high school”. The regression analysis was conducted in 4 steps. In step 1, the variable of place of residence was added. In step 2, the CAWAI-sum was added. The goal orientations mastery- and performance orientation were added in step 3, and intrinsic motivation was added in step 4 of the model. Significance levels were set to *α* < .050 for all statistical analyses. All analyses were performed using IBM SPSS (version 25).

## 3. Results

### 3.1. Descriptive statistics

The athletes in the current study rated “a natural choice” (3.79 ± 1.11), “priority of education or work” (3.61 ± 1.30), “lack of motivation” (3.49 ± 1.28), “negative performance development” (3.46 ± 1.18), and “challenges with health” (injuries or illness; 3.25 ± 1.54) as the highest ranked fixed statements of causes for drop out in cross-country skiing. Whereas “Not optimal training facilities” (1.74 ± .89) was the lowest ranked statement followed by “lack of variation in competition program” (2.28 ± .90), “ineffective coach-athlete relationship” (2.38 ± 1.15), “ineffective sports environment” (2.41 ± 1.17), and “high demands to equipment and economic resources” (2.44 ± 1.32). Table 1 reports descriptive statistics for the variables in the current study.

**Table 1:** Presents Pearson's correlations, means, standard deviations, minimum and maximum scores, reliability coefficients (Cronbach's alpha) and number of items for each variable.

Variable	1	2	3	4	5	6	7	8	9	10
1. Active	–									
2. Performance group	.17	–								
3. CAWAI-bond	−.28^**^	−.19^*^	−							
4. CAWAI-task	−.24^**^	−.25^**^	.80^**^	−						
5. CAWAI-goal	−.26^**^	−.26^**^	.78^**^	.84^**^	−					
6. CAWAI-sum	−.28^**^	−.25^**^	.93^**^	.93^**^	.93^**^	−				
7. PAP	−.17	−.25^**^	.40^**^	.61^**^	.50^**^	.53^**^	−			
8. Mastery orientation	−.21^*^	−.19^*^	.62^**^	.61^**^	.57^**^	.64^**^	.43^**^	−		
9. Performance orientation	−.02	.03	−.15	−.02	−.10	−.10	.14	−.19^*^	−	
10. Intrinsic motivation	−.13	−.05	.26^**^	.24^**^	.25^**^	.27^**^	.14	.31^**^	.04	−
Mean	−	−	5.23	5.02	5.20	5.15	4.39	5.09	4.31	5.68
Standard deviation	−	−	1.37	1.10	1.16	1.13	1.45	.92	.94	1.10
Max. score	2	1	7	7	7	7	7	7	6.5	7
Min. score	1	0	1.25	1.75	1.25	1.42	1	2.75	1.75	2.25
Cronbach's alpha	−	−	.92	.82	.83	.94	.94	.70	.65	.89
N. items	1	1	4	4	4	12	4	4	4	4

^*^ *p* < .050.

^**^ *p* < .010; Computations based on cross-sectional data collected from 116 participants.

The subscales of the CAWAI share large positive internal correlations, and PAP share large correlations with subscales and sum variable of CAWAI. The mean value of intrinsic motivation among the athletes in the current study was high during the period at high school.

There were significant differences between active and dropped out in scores for CAWAI-bond (6.08 ± .91 vs. 5.07 ± 1.38; *p* = .001), CAWAI-task (5.61 ± .92 vs. 4.90 ± 1.09; *p* = .006), CAWAI-goal (5.87 ± .98 vs. 5.07 ± 1.15; *p* = .004), CAWAI sum (17.55 ± 2.59 vs. 15.04 ± 3.37; *p* = .001). There was also a significant difference found for mastery orientation between the group of active and dropped out athletes (22.11 ± 2.88 vs. 20.00 ± 3.74; *p* = .010).

There were no significant differences found between active and dropped out in the scores on the PAP measure (19.79 ± 7.20 vs. 17.08 ± 5.40; *p* = .134), and performance orientation (17.42 ± 3.47 vs. 17.23 ± 3.85; *p* = .828) groups, nor for the intrinsic motivation scores (24.05 ± 3.72 vs. 22.47 ± 4.48; *p* = .113). Descriptive statistics and corresponding *p*-values from the independent samples t-test for athletes who are still active as cross-country skiers and athletes who dropped out are shown in Table 2.

**Table 2:** Independent samples *t*-test with mean, standard deviation and *p*-values for the group of athletes who are still active (*n* = 19) and the group of athletes who had dropped out of sport (*n* = 97).

Variable	Active	SD	Dropped out	SD	P
	Mean		Mean		
CAWAI-bond	6.08	.91	5.07	1.38	0.001
CAWAI-task	5.61	.92	4.90	1.09	0.006
CAWAI-goal	5.87	.98	5.07	1.15	0.004
CAWAI sum	17.55	2.59	15.04	3.37	0.001
PAP	19.79	7.20	17.08	5.40	0.134
Mastery orientation	22.11	2.88	20.00	3.74	0.010
Performance orientation	17.42	3.47	17.23	3.85	0.828
Intrinsic motivation	24.05	3.72	22.47	4.48	0.113

### 3.2. Variables predictive of drop-out

A hierarchical binary logistic regression analysis was calculated to predict if athletes were still active or had dropped out of sport based on place of residence, the coach-athlete working alliance, mastery and performance goal orientation and intrinsic motivation. The results from the regression analyses are presented in Table 3.

**Table 3:** Hierarchical binary logistic regression analyses based on cross-sectional data collected from the 116 participants where drop-out is the dependent variable.

	Independent variables	B	SE B	Wald
Step 1	Place of residence	1.657	.776	4.56^*^
	*R* ^2^	.091		
Step 2	Place of residence	1.717	.801	4.60^*^
	CAWAI-sum	−.296	.104	8.04^**^
	*R* ^2^	.224		
Step 3	Place of residence	1.709	.799	4.57^*^
	CAWAI-sum	−.278	.133	4.40^*^
	Goal orientation-mastery	−.017	.098	.030
	Goal orientation-performance	−.029	.073	.16
	*R* ^2^	.226		
Step 4	Place of residence	1.730	.803	4.64^*^
	CAWAI-sum	−.267	.134	3.95^*^
	Goal orientation-mastery	−.007	.101	.01
	Goal orientation-performance	−.031	.073	.18
	Intrinsic motivation	−.047	.074	.40
Model	*R* ^2^	.232		

B, unstandardized regression coefficient; SE B, coefficient standard error; SB, standardized coefficient beta.

^*^ *p* < .050.

^**^ *p* < .010.

The total binary logistic regression model had good fit to the data [*χ*^2^ 17.05 (*p* = .004)], and indicates that place of residence and the coach-athlete working alliance were significant predictors of drop out from cross-country skiing [*χ*^2^ = 17.05, df = 5 and *p* = .004 (<.05)]. The other possible predictors, goal orientation mastery and performance, and intrinsic motivation were not significant and had small effect on *R*^2^ when added, 002 and .006 respectively. All five predictors explain 23% of the variability in drop out from cross-country skiing. In step 1, place of residence was entered in the model and explains 9% of the variability in drop out from cross-country skiing. When CAWAI-sum was entered in step 2 in the model the variables explain 22% of the variability in drop out from cross-country skiing, and when goal orientation was entered in step 3, the variables explain 23% of the variability in drop out from cross-country skiing. Place of residence and CAWAI-sum are significant at the 0.05 level at the final step 4 [Place of residence Wald = 4.64, *p* = .031 (*p* < 0.05); CAWAI-sum Wald = 3.95, *p* = .047 (*p* < 0.05)]. The odds ratio (OR) for place of residence is 5.64 (95% CI: 1.170–27.189) and for CAWAI-sum the corresponding figures are .766 (95% CI: .589–.996). The model correctly predicted 11% of the cases where athletes are still active in cross-country skiing and 98% of the cases where athletes had dropped out of cross-country skiing, giving an overall percentage correct prediction rate of 84%.

### 3.3. Elite and general cross-country skiing groups

There were significant differences in scores for CAWAI-bond for the elite (5.57 ± 1.19) and the general group (4.83 ± 1.4; *p* = .004, two-tailed), for CAWAI-task for the elite (5.33 ± 1.06) and general group (4.67 ± 1.06; *p* = .001, two tailed), for CAWAI-goal for the elite (5.56 ± 1.10) and the general group (4.78 ± 1.10; *p* = .000, two-tailed), and for CAWAI sum for the elite (14.46 ± 3.10) and for general group (14.28 ± 3.37; *p* = .000, two-tailed). There was also a significant difference found for mastery orientation between the elite group (21.19 ± 3.50) and the general group (19.36 ± 3.66; *p* = .008, two-tailed), and between the PAP scores for the elite group (19.17 ± 5.98) and the general group (15.69 ± 5.19; *p* = .001, two-tailed).

There were no significant differences found in the scores on the performance orientation between the elite (17.24 ± 3.84) and general group (17.18 ± 3.76; *p* = .934, two-tailed), nor for the intrinsic motivation scores between the elite (23.00 ± 4.43) and the general group (22.44 ± 4.40; *p* = .499, two-tailed). The results can also be found in Table 4.

**Table 4:** Independent samples *t*-test with mean, standard deviation and *p*-values for the elite group of athletes (*n* = 58) and general groups (*n* = 55) within cross-country skiing at high school.

Variable	Elite		General		P
	Mean	SD	Mean	SD	
CAWAI-bond	5.57	1.19	4.83	1.45	.004
CAWAI-task	5.33	1.06	4.67	1.06	.001
CAWAI-goal	5.56	1.10	4.78	1.10	.000
CAWAI sum	16.46	3.10	14.28	3.37	.000
PAP	19.17	5.98	15.69	5.19	.001
Mastery orientation	21.19	3.50	19.36	3.66	.008
Performance orientation	17.24	3.84	17.18	3.76	.934
Intrinsic motivation	23.00	4.43	22.44	4.40	.499

## 4. Discussion

The current study investigated drop-out from cross-country skiing based on data collected from three Norwegian high schools with both elite- and general programs. Specifically, the study explored wheter living residence, the coach-athlete working alliance, goal orientation, and intrinsic motivation could explain drop-out, and if elite groups in high school are preventive for drop-out. The main findings were as follows; (1) The cross-country skiers who were still active had stronger working alliances with their coaches and higher mastery orientation at their time in high school than the athletes who dropped out from sport. However, there were no significant differences in the athlete's perceived performance, performance orientation and intrinsic motivation between the two groups. (2) Living residence and coach-athlete working alliance significantly predicted drop-out from cross-country skiing, while mastery- and performance orientation as well as intrinsic motivation did not contribute to explain drop-out. (3) Athletes included in elite cross-country skiing programs during high school had stronger coach-athlete working alliances, higher perceived performances, and higher mastery orientation than the athletes included in general groups, while no significant differences in performance orientation and intrinsic motivation were found between the athletes included in elite and general groups.

### 4.1. The importance of a strong coach-athlete working alliance

The results in the current study showed that athletes who were still active in competitive cross-country skiing after high school reported significantly stronger working alliances with their coaches during their time in high school, than the athletes who had dropped out from cross-country skiing. Both the sum of the coach-athlete working alliance and all three subscales (bond, task and goal) were significantly higher among the athletes who were still active compared with the athletes who had dropped out. Accordingly, the results from the regression analysis showed that the coach-athlete working alliance uniquely and significantly predicted drop-out in cross-country skiing. Thus, a stronger coach-athlete working alliance during the time in high school may indicate a higher probability of continuing with cross-country skiing after high school. The current results further substantiates previous research that highlights the importance of the coach-athlete relationship, and how the relationship relates to both athletes' performances and their physical- and mental well-being (27–30). Previous studies have also shown that a strong working alliance is negatively associated with athlete burnout and worry, and therefore the coach-athlete relationship is also a crucial protective factor of athletes' negative experiences in sports (28, 31). There is also a moderate correlation between athlete's perceived performance and the coach-athlete working alliance in the current study. Importantly, the coach-athlete working alliance indirectly represents coaches' competencies within attention skills in communication and their competencies to influence the development of their athletes within sport-specific capacities and skills (25).

A possible explanation of why the coach-athlete relationship seems to be a key factor to drop-out in cross-country skiing may be related to the expectation athletes have for their coaches to really engage in the helping relationship to make them fulfill their talents. Athletes who attend high schools specialized for their respective sports normally engage in sport to develop their skills and talents maximally. Previous research have showed that these athletes often have high ambitions (25). The results from the fixed statements in the current study showed that prioritization of education and/or work, lack of performance development and lack of motivation were the most reported causes for dropping out of cross-country skiing. Interestingly, the athletes' intrinsic motivation related to cross-country skiing during high school was the highest scored variable in the current study, both in the still active group and the drop-out group. Thus, these results might indicate that the helping relationships for athletes who had dropped out of cross-country skiing after graduating from high school, did not meet their expectations of performance development that they had during their time in high school. The athlete's perceived performance at their time during high school did not reach statistical significance between the two groups (because of large SD), but the athlete's perceived performance scores were higher in the group that were still active in cross-country skiing (19.79 ± 7.20 vs. 17.08 ± 5.40, respectively). This might be a plausible explanation why the coach-athlete working alliance predicts drop-out in the current study, and why motivation towards other activities and loss of motivation towards cross-country skiing are reported reasons to drop-out from cross-country skiing. However, these possible associations must be further investigated in future research.

### 4.2. Living residence outside athletes' family homes might build resilience

There is currently a lack of research concerning living situations and drop-out in general, whether from sports or school. However, living alone can affect the mental and physical health (58), meaning in cases where individuals live alone their social support system may be extra important. In Norway, it is not that unusual for high school students to live away from home during high school. Most students live alone in a small, rented apartment, while some schools offer dorm-style solutions for their students to live in together with other students. Previous research has been done on the effects living alone has on your mental and physical health (56), as well as the negative effects it can have on dietary habits and amount of physical exercise (59). The second hypothesis in the current study showed that place of residence was found to be a significant predictor of drop-out. In the first step of the regression analysis the variable place of residence was used to examine its association with drop-out. The results show that a place of residence outside the family home was a significant predictor of athletes still being active in competitive cross-country skiing, and the variable explained uniquely 9% of the variance. Thus, the results in the current study show that if the athletes lived at a residence outside of their family during their time in high school, they had a raised probability to continue with cross-country skiing after graduating from high school.

Becoming as good as possible may lead some highly motivated athletes to attend sport-specific schools at high school even when these are far away from their family residence (60), For elite athletes who are under a lot of pressure, not just with studies, but also high-performance training and competitions, their mental and physical health (including their diet) is paramount. Alas, high school students living on their own trying to combine studies and high-performance training and competitions, may end up feeling immense amounts of pressure and be overwhelmed by the number of extra tasks they can face living alone. Considering the results of the current study however, the athletes who faced these additional new challenges were not more likely to drop-out. On the contrary, learning to handle these additional responsibilities, might be important to build the athletes' character, independence, and resilience. With the proper guidance from coaches, teachers, and parents, these athletes can potentially become more autonomous, self-reliant and experience more self-ownership of their lives (61) than their peers who lived in the family residence. A study of young athletes attending sport schools in Germany similarly found that athletes who lived in boarding schools exhibited even better development of volitional skills, such as self-regulation and self-optimization, than those who lived with their parents (62, 63). Therefore, a possible explanation of the current study's results is that the athletes who lived away from their family residence became better prepared for the life of an athlete after high school and going into their senior careers.

### 4.3. The importance of mastery orientation in the development process

The significant difference found in mastery orientation between the active athletes and those who have dropped out seems to align with research which has found that task-involved individuals perceiving mastery criteria find optimized motivation, higher task-investment, longer persistence, higher performance and higher satisfaction, enjoyment and general positive regard about themselves and the task (43, 48). Mastery goals focus more on how a skill is performed or on the mastering of new skills, techniques or capacities (64) than purely on the outcome or result. Since this orientation is more focused on the process of learning, and doing one's best, scoring higher could potentially mean the athletes put less pressure on their results and leave more room for mistakes to occur. Interestingly, the highest correlations in the current study is between mastery orientation and the coach-athlete working alliance sum and the three subscales (.64, .62, .61, and .57), and mastery orientation is significantly higher among the athletes who were in elite groups during high-school. Thus, a strong working alliance between coaches and their athletes is associated with mastery orientation.

On the other hand, our study found no significant differences between active athletes and those who had dropped out in terms of performance goal orientation. Performance goals concerns an athlete's performance to a specific outcome or result (45, 46, 54), and most athletes who attend sport-specific high school programs in Norway have high ambitions (23). However, the current results indicate that the athletes in general have a stronger focus on the process of learning (mastery goals) on their path toward goal achievement. Cervello and colleagues (48) found that a high focus on outcome and results (performance goals) and a low perception of ability can be a predictor of drop-out behavior. However, when the goal orientations were added in the third step of the present study's regression analysis, they did not show a significance in predicting drop-out.

Furthermore, in previous reviews on the drop-out phenomenon in sports, several studies have found motivation to be an influencing factor (18, 19) in drop-out and drop-out behavior. However, the current study found no significant differences between active athletes and those who had dropped-out from cross-country skiing in terms of their intrinsic motivation during their time at high school. Thus, the athletes' intrinsic motivation at their time during high school did not predict drop out in the current study. One explanation may be that athletes who have chosen to attend to a sport-specific program in high-school may be highly motivated in general, and for these athletes, motivation on its own was therefore not a deciding factor of drop-out in cross-country skiing. In the present study, intrinsic motivation was the highest scored variable, indicating that this was quite a homogenous group with little variation in terms of intrinsic motivation.

### 4.4. Elite groups have stronger coach-athlete working alliances with their athletes

The third hypothesis was partially confirmed as the elite cross-country skiing group investigated scored higher on the coach-athlete working alliance, mastery orientation and perceived performance in comparison to the general group. However, performance orientation and intrinsic motivation scores were not significantly different between the two groups. The difference in reported coach-athlete working alliances between the participants in the elite and general groups was significant, which means that the athletes in the elite groups believed that their coaches actions and behaviors were more reliable to establish trust and to achieve progress in their sport. Thus, the results in the current study give reason to indicate that the coaches in the elite groups were perceived by their athletes to be more competent than their peers in the general groups. This argument has support in earlier research (65, 66). However the cause of such a difference may be a question for future research to investigate. There was also a significant difference found in the perceived athlete performance variable between the athletes in the elite group and those in the general group. This may indicate that the athletes in the elite group did in fact perform better than those in the general group, but perceived performance seemingly did not influence whether athletes were still active or not. This finding might support the research of Crane and Temple (8) who found feelings related to lack of coach competence being the second most cited reason for drop-out in their literature review. It could be interesting to conduct further research to see if participation in elite groups, or programs, may work as a protective factor regarding drop-out from sports.

### 4.5. Conclusion and limitations

The results in the current study highlight the importance of a strong coach-athlete working alliance in order to prevent athletes from dropping out of cross-country skiing, and that a living residence outside the family home might be preventive for drop-out from cross-country skiing after graduating from high school. The coaches in elite groups in the current study build significantly stronger working alliances with their athletes, focus significantly higher on mastery goal orientation, and this group of athletes perceive that their performances are significantly better than the athletes in the general group. The optimal coach-athlete working alliance includes a close relationship, where there is established a commonness concerning an athlete's thoughts and feelings (bond), about what goals to pursue and related strategies that are experienced as effective in order to reach the defined goals (28). Thus, this is a challenging task for a coach, and it is key to invest time in the relationship to develop such an optimal relationship between coaches and their athletes. Based on the current study coaches are key to prevent drop-out from cross country skiing.

However, there are several limitations in the current study. Firstly, the retrospective data collection procedure, which asks participants to reflect on a time in the past, is an important limitation and possible recall bias. In the present study, this included the participants' memory of the coach-athlete relationship, their intrinsic motivation, goal orientation, and performances from their time during high school. The athletes in the current study completed the questionnaire between 2 and 6 years after graduating from high school and if data was collected during their graduating years, the results may have been different. Memory can deteriorate or change over time due to various factors, and therefore the fact that the participants responded years after finishing high school may influence their subsequent responses. Furthermore, this study consisted of self-report measures which may also have an impact on the current results, as some research consider such measures as a fallible source of data (67). The full questionnaire had up to 90 questions in total and therefore potential respondent fatigue may become a factor influencing responses in the later items of the questionnaire (68). Additionally, approximately 50% of the participants in the current study did not report their biological sex. This is a limitation of the study since investigating potential differences between sexes were not possible. Previous researchers have found small differences in the perceived importance of the coach-athlete relationship and how it associates with drop-out behavior in female compared to male athletes (20). Lastly, another limitation of the study is the size of the sample and the fact that 50% of the invited participants did not reply to the invitation to participate in the current study. The analyses are conducted on only 50% of the potential sample. This potential selection bias must be acknowledged when considering the implications of the results found in the present study. The current study examined the perspective of elite high school athletes, and future research should also include the coach's perspective to get a more complete view on the coach-athlete working alliance. Furthermore, using a measure of coaching competency and/or efficacy may be useful for a more complete picture of the relationship. This study highlights the potential need for further focus on coaching competency and training, specifically concentrating on how to develop and maintain an effective and successful coach-athlete working alliance.

## Data Availability

The raw data supporting the conclusions of this article will be made available by the authors, without undue reservation.
